# Activation of the G Protein-Coupled Estrogen Receptor Elicits Store Calcium Release and Phosphorylation of the Mu-Opioid Receptors in the Human Neuroblastoma SH-SY5Y Cells

**DOI:** 10.3389/fnins.2019.01351

**Published:** 2019-12-17

**Authors:** Xiaowei Ding, Ting Gao, Po Gao, Youqiang Meng, Yi Zheng, Li Dong, Ping Luo, Guohua Zhang, Xueyin Shi, Weifang Rong

**Affiliations:** ^1^Department of Anesthesiology, Xin Hua Hospital, School of Medicine, Shanghai Jiao Tong University, Shanghai, China; ^2^Department of Anatomy and Physiology, Faculty of Basic Medical Sciences, School of Medicine, Shanghai Jiao Tong University, Shanghai, China; ^3^Department of Neurosurgery, Xin Hua Hospital Chongming Branch, School of Medicine, Shanghai Jiao Tong University, Shanghai, China

**Keywords:** estrogens, G protein-coupled estrogen receptor, calcium mobilization, μ opioid receptor, protein kinase C, protein phosphorylation

## Abstract

Estrogens exert extensive influences on the nervous system besides their well-known roles in regulation of reproduction and metabolism. Estrogens act via the nuclear receptor ERα and ERβ to regulate gene transcription (classical genomic effects). In addition, estrogens are also known to cause rapid non-genomic effects on neuronal functions including inducing fast changes in cytosolic calcium level and rapidly desensitizing the μ type opioid receptor (MOR). The receptors responsible for the rapid actions of estrogens remain uncertain, but recent evidence points to the G protein-coupled estrogen receptor (GPER), which has been shown to be expressed widely in the nervous system. In the current study, we test the hypothesis that activation of GPER may mediate rapid calcium signaling, which may promote phosphorylation of MOR through the calcium-dependent protein kinases in neuronal cells. By qPCR and immunocytochemistry, we found that the human neuroblastoma SH-SY5Y cells endogenously express GPER and MOR. Activation of GPER by 17β-estradiol (E2) and G-1 (GPER selective agonist) evoked a rapid calcium rise in a concentration-dependent manner, which was due to store release rather than calcium entry. The GPER antagonist G15, the PLC inhibitor U73122 and the IP3 receptor inhibitor 2-APB each virtually abolished the calcium responses to E2 or G-1. Activation of GPER stimulated translocation of PKC isoforms (α and ε) to the plasma membrane, which led to MOR phosphorylation. Additionally, E2 and G-1 stimulated c-Fos expression in SH-SY5Y cells in a PLC/IP3-dependent manner. In conclusion, the present study has revealed a novel GPER-mediated estrogenic signaling in neuroblastoma cells in which activation of GPER is followed by rapid calcium mobilization, PKC activation and MOR phosphorylation. GPER-mediated rapid calcium signal may also be transmitted to the nucleus to impact on gene transcription. Such signaling cascade may play important roles in the regulation of opioid signaling in the brain.

## Introduction

Estrogens exert an extraordinarily wide spectrum of actions in the human body. Besides the well-known roles in regulation of reproduction and metabolism, estrogens also exert multifaceted influences on neuronal development and neuronal functions (Jensen and Desombre, 1973). Traditionally, estrogens are known to act by interacting with two nuclear receptors, ERα and ERβ, which function as ligand-activated transcription factors to regulate gene transcription (Jensen and Desombre, 1973; Kuiper et al., 1996; Mosselman et al., 1996; Paech et al., 1997). In addition to this slow genomic mode of actions which typically develop with latencies ranging from an hour to several days, estrogens also directly alter neuronal electrical activity in various brain regions within seconds to minutes, which may underlie the fast effects of estrogens on brain functions such as female reproductive behavior, memory and cognition, neuroprotection and pain (Woolley, 1999; Kelly and Ronnekleiv, 2009; Ogawa et al., 2018).

Although the existence of the non-genomic estrogenic actions is now widely accepted, the mechanisms (the receptors and the signaling cascades) that mediate such effects remain uncertain and much debated. A number of candidate receptors have been proposed, including the classical ERα that may alternatively be bound to the plasma membrane, several ERα variants (ERα-52, ERα-46, and ERα-36), membrane-associated ER-X, the Gα_q_-coupled mERs and more recently the G protein-coupled estrogen receptor (GPER, also known as GPR30) (Rainville et al., 2015).

G protein-coupled estrogen receptor reportedly is enriched in discrete regions of the nervous system, including the hypothalamus, the hippocampus, the cerebral cortex, the dorsal horn of spinal cord and the primary afferent neurons, therefore is ideally positioned to mediate the rapid non-genomic estrogenic actions on reproductive behavior, memory and cognition and pain (Shughrue et al., 1997, 2000; Brailoiu et al., 2007; Kuhn et al., 2008; Dun et al., 2009; Hazell et al., 2009; Takanami et al., 2010; Lu et al., 2013). Indeed, numerous recent studies underscore the role of GPER in mediating the rapid estrogenic effects in the nervous system. For examples, intracerebroventricular infusion of the GPER agonist G-1 rapidly facilitates the female sexual behavior in estradiol-primed rats (Long et al., 2014, 2017). Activation of GPER in the dorsal hippocampus enhances social and object recognition and memory in the rat within 40 min (Paletta et al., 2018). In spinal cord slice *in vitro*, G-1 directly depolarizes superficial dorsal horn neurons; *in vivo*, intrathecal application of G-1 results in pain-related behaviors characterized by caudally directed scratching, biting and licking (Deliu et al., 2012).

Concerning the signaling cascades of the non-genomic effects, it occurs that estrogens may mediate rapid calcium signaling in neuronal and non-neuronal cells by modulating Ca^2+^ influx or inducing store Ca^2+^ release (Picotto et al., 1999; Rubio-Gayosso et al., 2000; Huang and Jan, 2001; Chen et al., 2002; Kuo et al., 2010; Petrovic et al., 2011). Thus, 17β−estradiol (E2) induces rapid Ca^2+^ influx in hippocampal neurons through activation of L-type Ca^2+^ channels, which probably mediates estrogen-induced neuroprotection (Wu et al., 2005, 2011; Zhao et al., 2005). E2 was also found to induce rapid Ca^2+^ release from intracellular Ca^2+^ stores in hypothalamic astrocytes and in embryonic midbrain dopaminergic neurons (Beyer and Raab, 1998; Kuo et al., 2010). In most cases, the receptor(s) responsible for the rapid Ca^2+^ rise was not clear, but recent evidence indicate that GPER may mediate estrogen-induced Ca^2+^ signaling. Revankar et al. (2005) expressed GPER as a fusion protein with green fluorescent protein (GFP) in COS7 cell (monkey kidney fibroblast) and found that activation of GPER resulted in intracellular Ca^2+^ mobilization and synthesis of phosphatidylinositol 3,4,5-trisphosphate in the nucleus. Incidentally, GPER is enriched in the hypothalamic-pituitary axis and the hippocampal formation (Paletta et al., 2018), where E2 has been reported to elicit cytosolic Ca^2+^ changes. Nevertheless, whether GPER mediates rapid Ca^2+^ signaling in neuronal cells is still uncertain.

Intracellular Ca^2+^ as a second messenger may activate protein kinases such as PKC and PKA to phosphorylate downstream effector proteins, which plays fundamental roles in neuronal signaling. Interestingly, E2 was found to rapidly attenuate the ability of mu-opioids to hyperpolarize hypothalamic neurons by uncoupling the μ-opioid receptors (MOR) from activating G protein-regulated inward rectifying potassium (GIRK) channels (Lagrange et al., 1997). Similarly, E2 uncouples other Gi/o-GPCRs, sepcifically GABA_B_ and opioid receptor-like (ORL)-1, from activating GIRK channels and the effect was dependent upon activation of PLC, PKA, and PKC (Conde et al., 2016). Therefore, estrogens appear to mediate a Ca^2+^-dependent phosphorylation and desensitization of Gi/o-coupled GPCRs through activating PKC and PKA. Whether GPER can initiate such non-genomic estrogenic signaling cascades remains to be determined.

The current study aims to test the hypothesis that GPER may mediate rapid Ca^2+^ signaling and subsequent Ca^2+^-dependent phosphorylation of MOR through activation of PKC in the human neuroblastoma SH-SY5Y cell line. Indeed, we found that SH-SY5Y cells endogenously express GPER and MOR and activation of GPER rapidly stimulates PLC/IP3-dependent store Ca^2+^ release with subsequent PKC activation and MOR phosphorylation.

## Materials and Methods

### Chemicals

G protein-coupled estrogen receptor agonist G-1 and GPER antagonist G-15 were purchased from Cayman Chemical Company (Ann Arbor, MI, United States). The pan-PKC inhibitor Ro 31-8820 was bought from ApexBio Technology Company (Houston, TX, United States). 17β-estradiol (E2) and other reagents were purchased from Sigma-Aldrich (St. Louis, MO, United States) unless otherwise mentioned.

### Cell Culture

The human neuroblastoma cell line, SH-SY5Y, was purchased from the Cell Repository of Chinese Academy of Sciences (Shanghai, China). The murine neuroblastoma Neuro-2a (N2A) cells (a wild type line and N2A cells stably expressing human influenza virus HA, YPYDVPDYA, epitope-tagged MOR) were provided by Dr. Yu Qiu (Shanghai Jiao Tong University School of Medicine). Cells were cultured in Dulbecco Modified Eagle Medium (DMEM), supplemented with 10% (v/v) fetal bovine serum (FBS), 100 units/ml penicillin, 100 μg/ml streptomycin, 0.11 g/L sodium pyruvate and 2 mM glutamine. 250 μg/ml G418 was added into the medium for Neuro-2a cell line stably expressing human MOR (N2AMT). Cells were incubated at 37°C in a humidified atmosphere containing 5% CO_2_.

### Western Blot Analysis

Cells cultured to 80% confluence were collected and placed in RIPA buffer containing protease inhibitors and phosphatase inhibitors to extract total proteins. Protein concentration was determined by BCA assay (Pierce, Rackford, IL, United States). We used the Biotin-Avidin-System to extract plasma membrane proteins. Briefly, SH-SY5Y cells were washed in phosphate-buffered saline (PBS) and subsequently incubated with Sulfo-NHS-LC-biotin (250 μg/ml in PBS) for 30 min and then with 10 mM glycine counteracted superfluous biotin for 20 min at 4°C. After extraction of total protein, NeutrAvidin Agarose Beads (Thermo Scientific, CN, United States) were added to the whole-cell lysates and incubated on rotating mixer for 3 h at 4°C. The mixture was centrifuged at 10,000 *g* for 30 min at 4°C. Subsequently, the beads were washed for five times and the plasma membrane proteins were eluted and denatured by 2 × SDS-PAGE sample loading buffer at 100°C for 5 min. 25 μg of total proteins or 30 μl sample loading buffer containing plasma membrane proteins were electrophoresed on 4–8% Tris-glycine ready gels (Bio-rad, Hercules, CA, United States). The separated proteins were transferred from the gel to the surface of nitrocellulose membranes (Bio-rad). The membranes were blocked with 5% fat-free dry milk or 5% BSA (for detection of phosphorylated MOR, PKCα, Na^+^-K^+^-ATPase) in Tris-buffered saline (TBS) containing 0.1% Tween-20 for 2 h. Subsequently, the membranes were incubated with primary antibodies for 18 h at 4°C: rabbit GPER (1:1000, Abcam, Cat# ab39742, RRID:AB_1141090), rabbit anti-pMOR (1:1000, Cell Signaling Technology, Cat# 3451, RRID:AB_331619), rabbit anti-MOR (1:500, Novus, Cat# NBP1-31180, RRID:AB_2251717), rabbit anti-PKCα (1:1000, Cell Signaling Technology, Cat# 2056, RRID:AB_2284227), mouse anti-PKCε (1:1000, BD Biosciences, Cat# 610085, RRID:AB_397492), rabbit anti-Na^+^-K^+^-ATPase (1:3000, Abcam, Cat# ab76020, RRID:AB_1310695) and mouse anti-β-actin (1:2000, Bioworld Technology, BS6007M). Bound primary antibodies were detected with HRP-conjugated anti-rabbit (1:3000, Bio-Rad, Cat# 170-6515, RRID:AB_11125142) or anti-mouse (1:3000, Bio-Rad, Cat# 170-6516, RRID:AB_11125547) secondary antibody. Immunoreactive bands were visualized using enhanced chemiluminescence (Thermo, Indianapolis, IN, United States), and digital imaging was captured with an Image Quant LAS 4000 mini (GE Healthcare, Life Science). The density of specific bands was analyzed using NIH ImageJ software and was normalized against the loading controls (β-actin, GAPDH or Na^+^-K^+^-ATPase).

### Immunofluorescence Staining

SH-SY5Y cells were seeded on glass coverslips and cultured for 24 h and fixed with 4% paraformaldehyde for 15 min. After washing with PBS, the cells were first incubated with 50 mM PBS containing 10% normal goat serum and 0.5% TritonX-100 at room temperature for 2 h to block non-specific binding and this was followed by incubation with rabbit anti-GPER (1:500, Abcam, Cat# ab39742, RRID:AB_1141090) or rabbit anti-MOR (1:500, Novus, Cat# NBP1-31180, RRID:AB_2251717) at 4°C overnight. The cells were rinsed with PBS for four times and were then incubated with goat anti-rabbit Alexa fluor 568 (1:1000; Molecular Probes-Invitrogen, Cat# A-11077, RRID:AB_141874) or 488 (1:1000; Molecular Probes-Invitrogen, Cat# R37116, RRID:AB_2556544) secondary antibody at room temperature for 1.5 h. GPER or MOR were counter-stained with a nuclear marker DAPI (1: 1000, Thermo Fisher Scientific, Cat# PA5-62248, RRID:AB_2645277) at room temperature for 10 min. The coverslips were mounted on glass slides and the cells were viewed under the fluorescent microscope (Leica DM2500, Leica Microsystems Limited).

### Real-Time Reverse Transcription-Polymerase Chain Reaction (RT-PCR)

Total RNA of SH-SY5Y and Neuro-2a cells was extracted with Trizol (Invitrogen, Shanghai, China) according to the manufacturer’s instructions and reversely transcribed into cDNA using oligo-dT primers. Real-time quantitative PCR was then performed using SYBR Green (Qiagen, Shanghai, China) as the reporter dye. All cDNA samples were analyzed in duplicate. The relative level of target mRNA was calculated by the method of 2^–Δ^ ^Δ^ ^Ct^ with GAPDH as the loading control. The primer sets for real-time PCR are as follows:

GPER (human): Forward 5′-TCACGGGCCACATTG TCAACCTC-3′ and Reverse 5′-GCTGAACCTCACATC CGACTGCTC-3′;

GAPDH (human): Forward 5′-GGAGCGAGATCCC TCCAAAAT-3′ and Reverse 5′-GGCTGTTGTCATACTTC TCATGG-3′;

GPER (mouse): Forward 5′-CCTCTGCTACTCCCT CATCG-3′ and Reverse 5′-ACTATGTGGCCTGTC AAGGG-3′;

GAPDH (mouse): Forward 5′-TGTCTTCACCACCAT GGAGA-3′ and Reverse 5′-CGGCCATCACGCCAC AGCTT-3′.

### Calcium Imaging

Cells were incubated with 1 μM Fluo-4-AM (Molecular Probes-Invitrogen) and 0.01% pluronic (Sigma-Aldrich) in the extracellular solution (NaCl 136 mM, KCl 5.4 mM, MgCl_2_ 1 mM, CaCl_2_ 1.8 mM, HEPES 10 mM, Glucose 10 mM, and NaH_2_PO_4_ 0.33 mM, pH7.4, osmotic pressure 300 mOsm/L, MgCl_2_ replaced CaCl_2_ for calcium free solution) at 25°C for 1 h. The cells were continuously superfused with the extracellular solution and imaged using an inverted microscope (Leca DMI4000B). Drugs (or vehicle control) were applied through a micro-perfusion tube positioned to the vicinity of the cells in the field of view. Fluorescent signal was excited at 510 nm and acquired at 580 nm, and taken every 3 s through a CCD camera. The signal was monitored online and analyzed offline, using Leica AF6000 software (Leica). The fluorescent traces were calculated as:

$$\%\mathrm{change}\mathrm{in}\mathrm{fluorescence}=\frac{\text{F-F}\,0}{\mathrm{F0}}\times 100$$

(F0: the baseline fluorescence of cells before treatments, F: the fluorescence of cells with drugs treatments).

### Statistical Analysis

Data are expressed as mean ± SEM. Statistical analysis was performed using GraphPad Prism 6 (GraphPad Software Inc., United States). Differences between two groups were analyzed by unpaired or paired *t*-test (two-tailed). Multiple comparisons were made by one-way analysis of variance (ANOVA) with Tukey’s *post hoc* testing. Differences were considered statistically significant when a *P* value was less than 0.05.

## Results

### SH-SY5Y Cells Endogenously Express GPER and MOR

**FIGURE 1:**
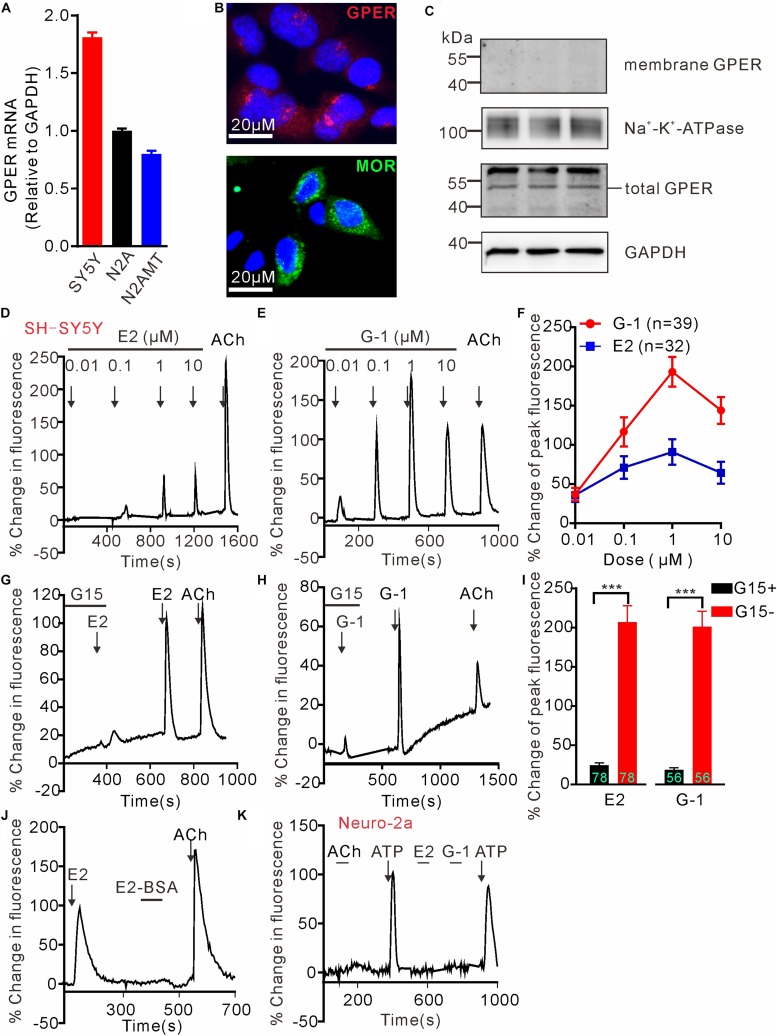
G protein-coupled estrogen receptor mediates calcium rise in SH-SY5Y cells. **(A)** RT-PCR analysis shows relatively higher expression of GPER mRNA in SH-SY5Y cells than in Neuro-2a (N2A) cells. N2AMT is a line of Neuro-2a stably expressing human μ type opioid receptor (hMOR). **(B)** Immunofluorescent staining for GPER and MOR in SH-SY5Y cells. GPER immunofluorescence is located at certain perinuclear organelle. **(C)** Western blot detection of GPER in cytoplasmic membrane fraction vs. total protein. Whereas GPER immunoreactivity is abundant in the total protein samples, it could not be detected within the cytoplasmic membrane protein sample. **(D–J)** Fluo-4-based imaging of cytosolic [Ca^2+^] transients in response to GPER agonists 17-β estradiol (E2) and G-1. Note E2 and G-1 both caused concentration-dependent rapid Ca^2+^ rise, which was prevented by GPER antagonist G15. The membrane impermeable BSA-conjugated E2 was unable to evoke a rapid Ca^2+^ rise. **(K)** Neither E2 nor G-1 was able to cause a Ca^2+^ rise in N2A cells. The example traces **(D**,**E**,**G**,**H**,**J**,**K)** are the average percentage change in fluorescence of 7–26 cells in one visual field. Values in bar graphs **(F**,**I)** are averaged peak percentage changes of 32∼78 cells from three to four independent tests. Note that the responses to the lowest concentration (0.01 μM) of E2 or G-1 in separate experiments were quite variable (e.g., **D,E**), but on average the peak response to E2 or G-1 (0.01 μM) were similar **(F)**. ^∗∗∗^*P* < 0.001, paired *t*-test.

G protein-coupled estrogen receptor mRNA expression in SH-SY5Y, wild type N2A and N2A cell line stably expressing human MOR (N2AMT) was analyzed through RT-PCR. GPER mRNA appeared to be more abundant in SH-SY5Y cells than in N2A and N2AMT cells (Figure 1A). Immunofluorescent staining confirmed SH-SY5Y cells endogenously express GPER and MOR (Figure 1B). GPER immunostaining was located at peri-nuclear sites. Western blot analysis detected GPER in total but not in cytoplasmic membrane protein samples (Figure 1C). Therefore, the endogenously expressed GPER in SH-SY5Y cells was located on certain peri-nuclear organelles, but not on the cytoplasmic membrane.

### GPER Mediates Rapid Intracellular Calcium Rise in SH-SY5Y Cells

To test the hypothesis that activation of GPER mediates rapid Ca^2+^ signaling, SH-SY5Y and the Neuro-2a cells were exposed to either 17β-estradiol (E2) or G-1, the latter being a GPER selective agonist that does not bind ERα or ERβ (Bologa et al., 2006). Brief exposure (30 s) of SH-SY5Y cells to increasing concentration of E2 or G-1 (0.01–10 μM) both produced fast and concentration-dependent increases in cytosolic Ca^2+^ levels (Figures 1D–F). Acetylcholine (ACh) was a positive control. Consistent with the intracellular localization of GPER in this cell line, the cell impermeable BSA-conjugated E2 (E2-BSA, 1 μM) was not effective (Figure 1J). In addition, the GPER selective antagonist G15 (Dennis et al., 2009) (3 μM) almost completely blocked the Ca^2+^ responses to E2 and G-1 (G15 + E2: 24.43 ± 3.10% vs. E2: 207.0 ± 20.70%, *P* < 0.001, *n* = 78 cells; G15 + G-1: 18.59 ± 2.79% vs. G-1: 201.0 ± 19.74%, *P* < 0.001, *n* = 56 cells, Figures 1G–I). In contrast to the SH-SY5Y cells, Neuro-2a cells did not respond to E2 or G-1 (1 μM) with a Ca^2+^ rise, consistent with the low GPER mRNA expression in this cell line (Figure 1K). It was noticed that Neuro-2a cells responded to ATP (100 μM) with a rapid Ca^2+^ rise but not to ACh. These results support the hypothesis that activation of GPER by E2 may mediate rapid Ca^2+^ signaling in neuroblastoma cells.

### GPER-Mediated Calcium Rise in SH-SY5Y Cells Is Due to Store Calcium Release

**FIGURE 2:**
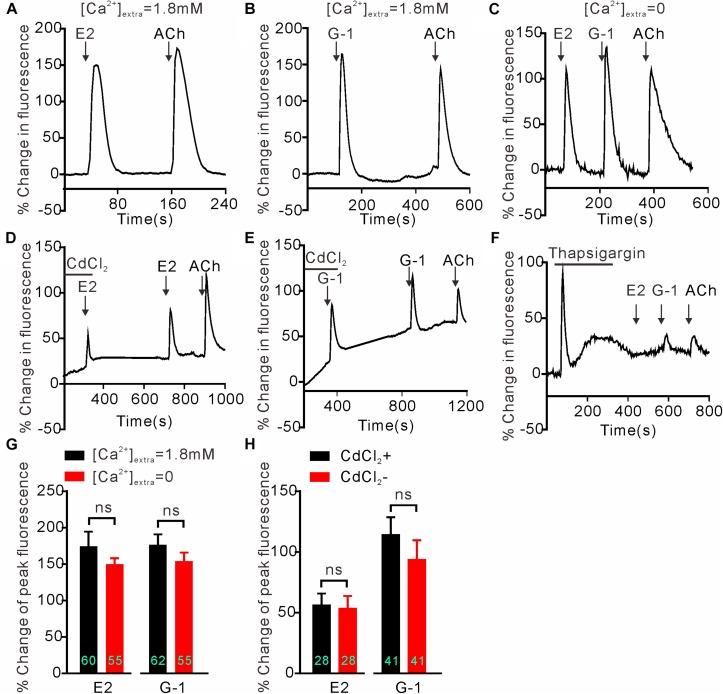
Intracellular calcium rise elicited by E2 or G-1 was independent of calcium influx in SH-SY5Y cells. **(A–C,G)** E2 and G-1 both caused rapid Ca^2+^ rise with or without extracellular Ca^2+^. **(D,E,H)** The non-selective voltage-gated calcium channel blocker CdCl_2_ had no effect on the Ca^2+^ rise induced by E2 or G-1. **(F)** Depletion of store Ca^2+^ using thapsigargin prevented E2 or G-1 or ACh from evoking a rapid Ca^2+^ rise. The example traces **(A–F)** are the average percentage change in fluorescence of 10–29 cells in one visual field. **(G,H)** Averaged peak percentage changes in fluorescence in different conditions. ns, not significant, unpaired **(G)** or paired **(H)** *t*-test, *n* = 28∼62 cells from three to four independent tests.

**FIGURE 3:**
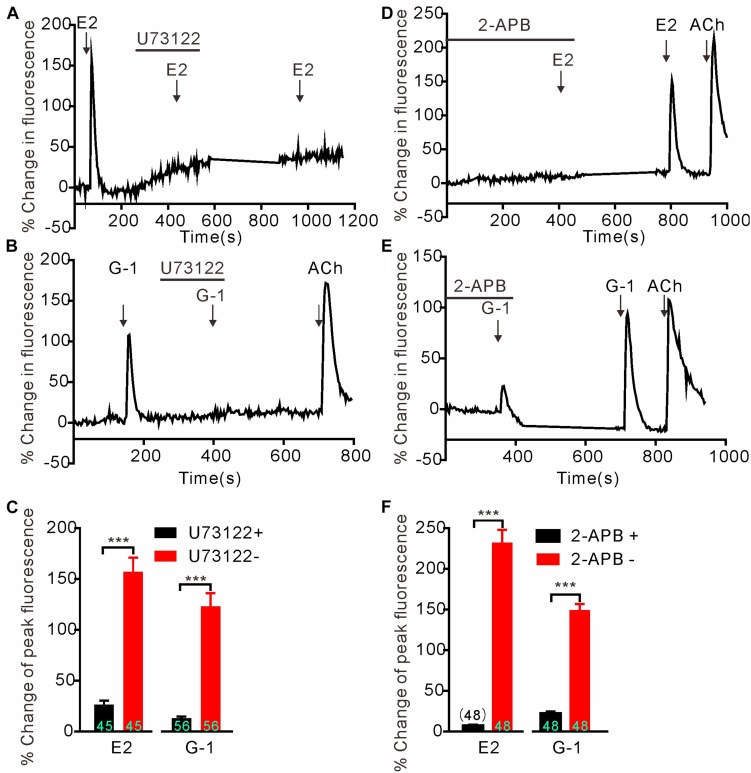
G protein-coupled estrogen receptor-mediated rapid calcium rise in SH-SY5Y cells is via the PLC/IP3 pathway. **(A–C)** The PLC inhibitor U73122 (3 μM) completely blocked the calcium response to E2 (1 μM) and G-1 (1 μM). **(D–F)** The store calcium release channel (IP3R) blocker, 2-APB, virtually abolished G-1 or E2-evoked calcium responses. The example traces **(A,B,D,E)** are the average percentage change in fluorescence of 16–25 cells in one visual field. **(C,F)** The averaged peak percentage changes in fluorescence in different conditions. ^∗∗∗^*P* < 0.001, paired *t*-test. *n* = 45∼56 cells from three to four independent tests.

We next investigated whether GPER-mediated Ca^2+^ rise is due to Ca^2+^ influx or store Ca^2+^ release. In the Ca^2+^-free extracellular solution, E2 (1 μM) and G-1(1 μM) both still elicited rapid increases in cytosolic Ca^2+^ with similar magnitudes as seen in the regular Ca^2+^-containing solution (Figures 2A–C) (E2 Ca^2+^-free: 154.1 ± 11.77%, *n* = 55 cells vs. E2 Ca^2+^-containing: 174.5 ± 20.09%, *n* = 60 cells, *P* > 0.05; G-1 Ca^2+^-free: 149.8 ± 8.40%, *n* = 55 cells vs. G-1 Ca^2+^-containing: 176.5 ± 14.34%, *n* = 62 cells, *P* > 0.05, Figure 2G), suggesting that the GPER agonists evoked store Ca^2+^ release rather than Ca^2+^ influx. In support, the broad-spectrum voltage-dependent Ca^2+^ channel blocker CdCl_2_ (200 μM) failed to block the increase in [Ca^2+^]_i_ elicited by E2 or G-1 (Figures 2D,E) (E2 + CdCl_2_: 56.24 ± 9.51% vs. E2: 53.38 ± 10.40%, *n* = 41 cells, *P* > 0.05; G-1 + CdCl_2_: 114.25 ± 14.50% vs. G-1: 93.66 ± 16.06%, *n* = 28 cells, *P* > 0.05, Figure 2H), whereas depletion of store Ca^2+^ with thapsigargin (1 μM, 5 min) virtually prevented E2 or G-1 from inducing a Ca^2+^ rise (Figure 2F). Furthermore, treatment of SH-SY5Y cells with the PLC inhibitor U73122 (3 μM) completely blocked the rapid Ca^2+^ rise in response to E2 or G-1 (Figures 2A,B, 3A–C). Analogously, E2 or G-1-induced Ca^2+^ rise was abolished in the presence of 2-APB (3 μM), an IP3R inhibitor (Figures 3D,E) (E2 + 2-APB: 7.12 ± 1.30% vs. E2: 231.5 ± 16.41%, *n* = 48 cells, *P* < 0.001; G-1 + 2-APB: 22.01 ± 2.73% vs. G-1: 148.9 ± 7.82%, *n* = 48 cells, *P* < 0.001, Figure 3F). Therefore, activation of GPER may elicit PLC/IP3-mediated store Ca^2+^ release.

### GPER Activation Stimulates PKC Isoforms

**FIGURE 4:**
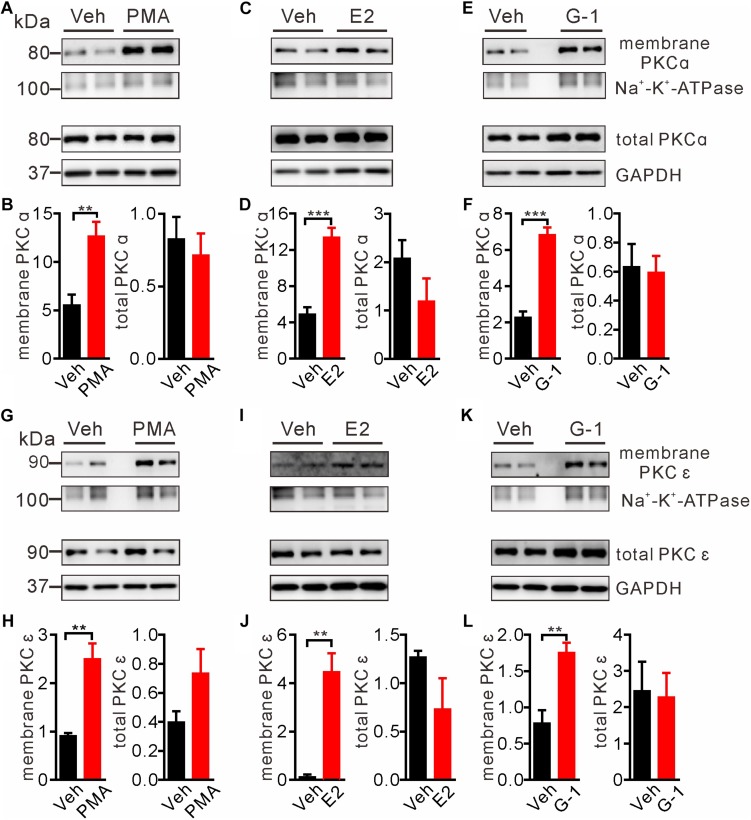
E2 and G-1 promoted translocation of PKC isoforms to the plasma membrane. **(A,B,G,H)** Cells treated with the pan-PKC agonist PMA (1 μM) for 5 min had significantly higher PKCα and PKCε in the plasma membrane protein samples, compared with vehicle-treated cells. **(C,D,I,J)** Cells treated with E2 (1 μM) for 5 min had increased PKCα and PKCε in the plasma membrane protein samples as compared with cells treated with vehicle. **(E,F,K,L)** Cells treated with G-1 (1 μM) for 5 min had increased PKCα and PKCε in the plasma membrane protein samples as compared with cells treated with vehicle. After detected PKCα bands **(C,E)**, the nitrocellulose membrane was stripped and re-blocked by 5% fat-free dry milk in TBST, followed by incubation with PKCε primary antibody, then PKCε bands was detected **(I,K)**. So the loading controls are the same. Membrane PKCα or PKCε is a relative value with Na^+^-K^+^-ATPase as the internal reference for plasma membrane protein; total PKCα or PKCε is the relative value with GAPDH as the internal reference for total protein. ^∗∗^*P* < 0.01, ^∗∗∗^*P* < 0.001, unpaired *t*-test, averaged data from three to four independent experiments.

Intracellular Ca^2+^ as an important second messenger may activate the Ca^2+^-dependent protein kinases (PKCs) characterized by translocation of cytosolic PKCs to the plasma membrane. Previous reports have implicated PKCα and PKCε in regulation of pain and μ-opioid signaling (Bailey et al., 2009; Zheng et al., 2011; Illing et al., 2014). Hence we focused on these two PKC isoforms to test whether GPER-mediated Ca^2+^ signaling may stimulate PKCs. We took advantage of the biotin-avidin method to extract plasma membrane proteins and to detect the possible translocation/activation of PKCα and PKCε following GPER activation in SH-SY5Y cells. As a positive control, cells treated with the PKC agonist PMA (1 μM) for 5 min manifested pronounced translocation of PKCα and PKCε to the plasma membrane (Figures 4A,B,G,H). In a similar manner, a 5 min treatment of the cells with E2 or G-1 (1 μM) significantly increased membrane translocation of PKCα and PKCε (Figures 4C–F,I–L).

### GPER Activation Facilitates MOR Phosphorylation in PKC-Dependent Manner

**FIGURE 5:**
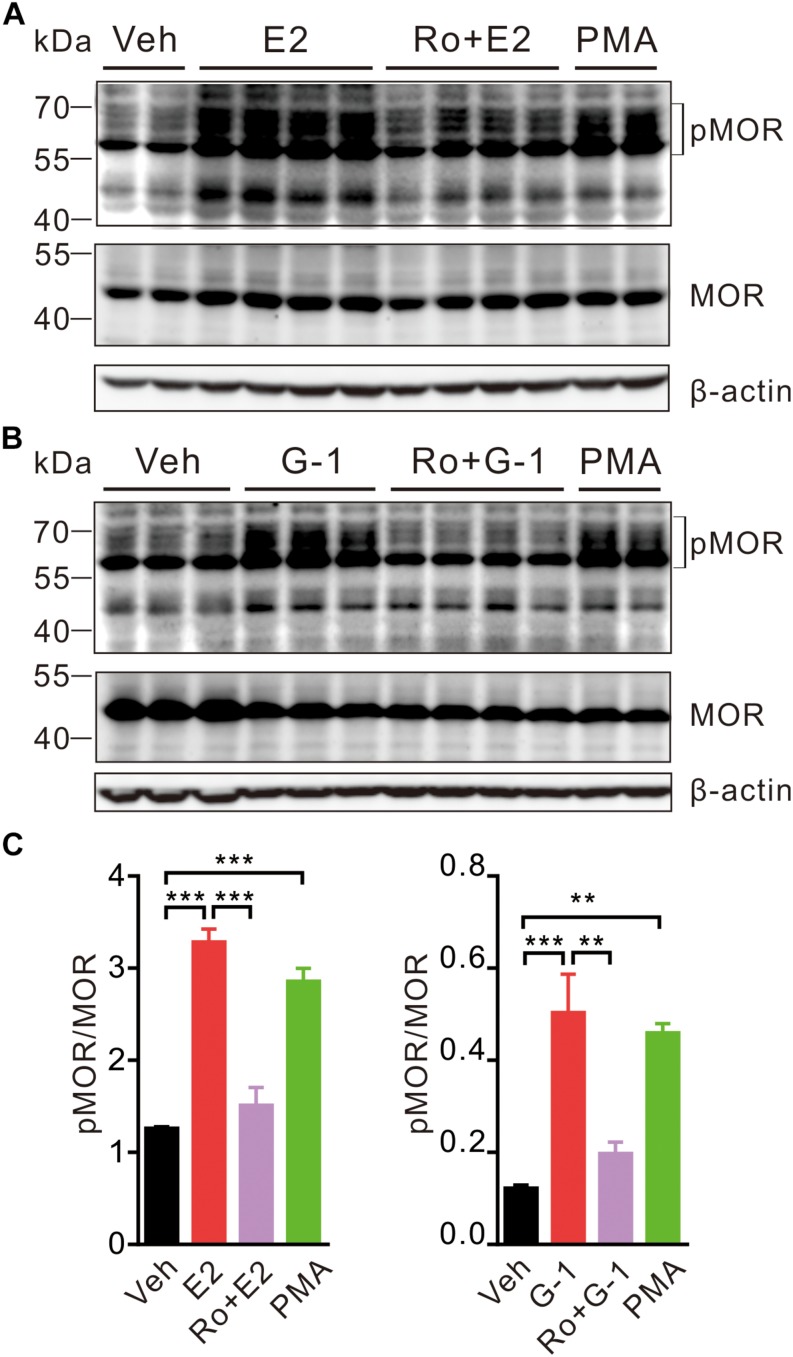
E2 and G-1 stimulate phosphorylation of MOR in a PKC-dependent manner. **(A,B)** SH-SY5Y cells treated with PMA (1 μM), E2 (1 μM), or G-1 (1 μM) for 30 min showed increased level of phosphorylated MOR (pMOR) as compared with the vehicle group. In the presence of the pan-PKC inhibitor (Ro 31-8820, Ro, 3 μM), E2 and G-1 failed to increase pMOR expression. **(C)** The averaged pMOR level (relative to β-actin) in cells with different treatments. After detection of pMOR **(A,B)**, the nitrocellulose membrane was stripped and re-blocked by 5% fat-free dry milk in TBST, followed by incubation with MOR primary antibody, then MOR band was detected. ^∗∗^*P* < 0.01, ^∗∗∗^*P* < 0.001, one-way ANOVA with Tukey’s *post hoc* test, averaged data from three to four independent experiments.

In the hypothalamus, estrogens may rapidly desensitize MOR probably by activating PLC, PKA, and PKCs and uncouple MOR from activating GIRK channels (Kelly et al., 1999). We therefore investigated whether activation of GPER may promote MOR phosphorylation in SH-SY5Y cells. As a positive control, cells were treated with the PKC agonist PMA (1 μM) for 30 min, which resulted in an increase of phosphorylated MOR (pMOR) level compared with the vehicle group (Figure 5). Similarly, cells exposed to E2 or G-1 (1 μM) also had significantly higher levels of pMOR, which was prevented by co-administration of pan-PKC inhibitor Ro 31-8820 (3 μM, which inhibits PKCα, PKCβI, PKCβII, PKCγ, and PKCε) (Figure 5). Therefore, activation of GPER may promote MOR phosphorylation in a PKC-dependent manner.

### GPER-Mediated Rapid Calcium Signaling Elicits Indirect Genomic Effects

**FIGURE 6:**
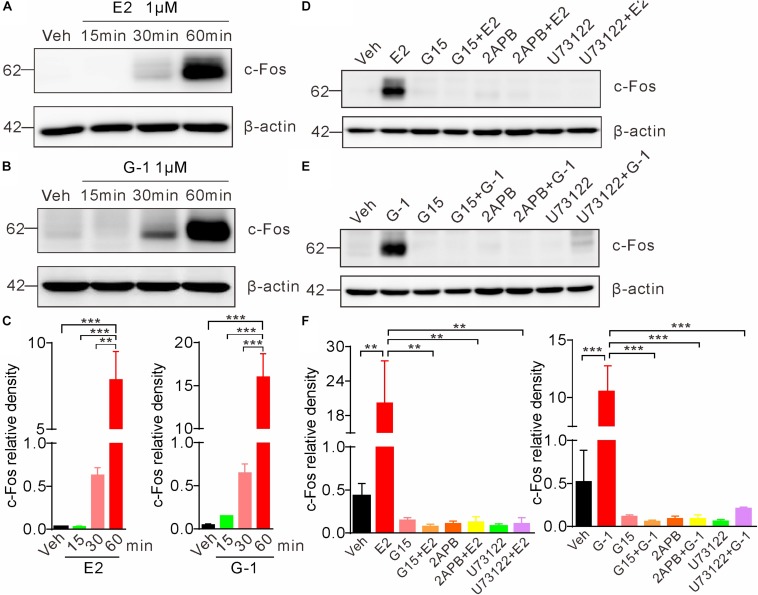
Activation of GPER induces c-Fos expression in SH-SY5Y cells. **(A–C)** Cells treated with E2 (1 μM) or G-1(1 μM) showed time-dependent increases in c-Fos expression. **(D–F)** E2 and G-1-induced c-Fos expression was prevented by the GPER antagonist G15 (1 μM), PLC inhibitor U73122 (1 μM), and IP3R inhibitor 2-APB (1 μM). c-Fos density was relative to β-actin. ^∗∗^*P* < 0.05, ^∗∗∗^*P* < 0.01, one-way ANOVA with Tukey’s *post hoc* test, averaged data from three independent experiments.

Previous studies indicate that the rapid non-genomic estrogenic signaling via second messengers (such as cAMP or Ca^2+^) is also transmitted to the nucleus to affect gene transcription and protein synthesis, leading to an indirect (not via the classic nuclear receptor ER α/β) genomic effect (Guo et al., 2002). To investigate whether GPER-mediated rapid Ca^2+^ signaling may elicit such indirect genomic effects, we exposed SH-SY5Y cells with E2 or G-1 and detected c-Fos, which is an immediate early gene that responds to extracellular signals. Cells were treated with E2 (1 μM) or G-1 (1 μM) for 15, 30, and 60 min, respectively. Whole lysate western blot showed that both E2 and G-1 caused time-dependent increases in c-Fos protein expression (Figures 6A–C). Moreover, the E2 or G-1-induced c-Fos expression was almost completely negated in the presence of the GPER antagonists G15, the IP3R inhibitor 2-APB, or the PLC inhibitor U73122 (Figures 6D–F). These results indicate that GPER-mediated rapid PLC/IP3-dependent Ca^2+^ signaling may be transmitted to the nucleus to regulate gene transcription and protein synthesis.

## Discussion

Estrogens, primarily 17β-estradiol (E2), are known to exert major influences on the nervous system with slow genomic and rapid non-genomic effects. Whilst it is clear that two nuclear receptors, ERα and ERβ, mediate the slow genomic effects, the receptor(s) responsible for the rapid non-genomic effects in the nervous system remain uncertain. The current study demonstrates that the human neuroblastoma SH-SY5Y cells endogenously express G protein-coupled estrogen receptor (GPER) and activation of GPER may induce a rapid increase in cytosolic Ca^2+^, which is due to PLC/IP3-dependent store Ca^2+^ release and is followed by PKC activation and MOR phosphorylation. Furthermore, GPER-mediated Ca^2+^ signaling may also be transmitted to the nucleus to induce c-Fos expression. To the best of our knowledge, this is for the first time that GPER is shown to mediate calcium release and PKC-dependent phosphorylation of MOR in neuroblastoma cells. Our findings strongly support GPER as a mediator of the rapid non-genomic estrogenic effects in the nervous system.

Ca^2+^ as a ubiquitous intracellular messenger regulates diverse neuronal functions. Ca^2+^ may bind to synaptotagmin to trigger synaptic vesicle release (Emptage et al., 2001). Ca^2+^ also binds to calmodulin (CaM) to form CaM-Ca^2+^ complex, activating Ca^2+^/CaM-dependent protein kinases (CAMKs) or the Ca^2+^/CaM-dependent serine/threonine phosphatase calcineurin, which plays an important role in the regulation of synaptic plasticity (Yakel, 1997; Wayman et al., 2008; Kim et al., 2016; Sugawara et al., 2017). Ca^2+^ activates the Ca^2+^-dependent protein kinase (PKC) system to promote phosphorylation of various GPCRs and ion channels, thereby regulating neuronal excitability, synaptic transmission and plasticity (Ritter et al., 2012; Fukuchi et al., 2015b; Li et al., 2017; Rajagopal et al., 2017; Summers et al., 2019).

Previous studies indicate that estrogens may induce rapid changes in cytosolic Ca^2+^ in hippocampal neurons, hypothalamic astrocytes and embryonic midbrain dopaminergic neurons (Brailoiu et al., 2007; Noel et al., 2009; Kuo et al., 2010). Furthermore, E2 was found to rapidly desensitize μ-opioid receptor (MOR) in hypothalamic neurons in PLC, PKA, and PKC-dependent manners, suggesting that Ca^2+^ signaling probably underlies estrogenic suppression of MOR function (Lagrange et al., 1997; Conde et al., 2016). MOR is best known for the regulation of pain and analgesia, but also plays important roles in regulation of reproductive behaviors, neuroprotection and cognition (Long et al., 2014; Jacobson et al., 2018; Liu et al., 2018; Vaidya et al., 2018). Therefore, it is of great interest to understand the identity of the receptor(s) which may mediate estrogenic suppression of MOR signaling.

The primary goal of this study is to test whether GPER can initiate rapid Ca^2+^ signaling and PKC-dependent phosphorylation of MOR in neuronal cells. To this end, we set to search for a neuronal cell line co-expressing GPER and MOR. qPCR showed that GPER mRNA appears to be relatively more abundant in the human neuroblastoma SH-SY5Y cells than in the murine Neuro-2a cells. Our immunofluorescent staining shows presence of GPER immunoreactivity at peri-nuclear sites and western blot assay could detect GPER immunoreactivity in whole-cell protein extract but not in membrane protein extract. Therefore, GPER is located intracellularly, in line with other reports indicating GPER being localized to the endoplasmic reticulum or the Golgi apparatus (Filardo and Thomas, 2005; Cheng et al., 2011). We found that GPER agonists E2/G-1 induced a rapid increase in intracellular calcium in SH-SY5Y cells but not in Neuro-2a cells. Currently, the reason for the lack of calcium response in Neuro-2a cells can only be speculated. One possibility is that GPER protein expression may be low in Neuro-2a cells, as suggested by the relative lower GPER mRNA than in SH-SY5Y cells. The other possibility is that GPER might be coupled to a different signaling cascade in this cell line. Due to the uncertain specificity of commercial GPER antibodies (in our hands, most GPER antibodies do not work very well with mouse samples), we have not been able to determine GPER protein expression in the murine-derived Neuro-2a cells. In this respect, it would be useful to test whether Neuro-2a cells transfected with GPER gene may respond to E2/G-1 with a calcium rise.

SH-SY5Y cells probably express ERα, ERβ, Gα_q_-mER as well GPER (Barbati et al., 2012; Mateos et al., 2012; Nakaso et al., 2014; Gray et al., 2016; Shen et al., 2016; Cheng et al., 2017). However, the following lines of evidence from this study indicate that activation of GPER but not the classical ERs or ER variants may initiate rapid Ca^2+^ signaling: (1) SH-SY5Y but not Neuro-2a cells responded to E2 and the GPER selective agonist G-1 with rapid rises in cytosolic Ca^2+^ in concentration-dependent manners, consistent with the relative abundance of GPER mRNA in this cell line; (2) Either E2 or G-1-induced Ca^2+^ rise was blocked by the GPER antagonist G15; (3) The membrane impermeable E2-BSA failed to cause a significant Ca^2+^ response, consistent with the intracellular localization of GPER in SH-SY5Y cells.

E2 has been shown to induce rapid Ca^2+^ influx through activation of L-type calcium channels (Wu et al., 2005, 2011; Zhao et al., 2005) or rapid store Ca^2+^ release in some neuronal cells (Beyer and Raab, 1998; Kuo et al., 2010). We found that GPER-mediated Ca^2+^ rise in SH-SY5Y cells was due to store release rather than Ca^2+^ entry since either E2 or G-1 still induced rapid increases in cytosolic Ca^2+^ in the extracellular Ca^2+^-free condition and in the presence of cadmium, a broad-spectrum voltage-dependent Ca^2+^ channel blocker. Depletion of the Ca^2+^ store with thapsigargin prevented E2 or G-1 from inducing a change in intracellular Ca^2+^ level. Furthermore, the IP3 receptor inhibitor 2-APB and the PLC inhibitor U73122 virtually abolished either E2 or G-1-induced Ca^2+^ response, confirming activation of GPER induces store Ca^2+^ release through PLC and IP3 pathway.

Previously, Grassi et al. (2015) reported that estrogen receptor modulator raloxifene may down-regulate vasopressin mRNA in SH-SY5Y cells. By using G-1, G-15 and a PKC inhibitor, authors concluded that the raloxifene effect was mediated by GPER and PKC. In the present study, we were able to confirm that GPER-initiated Ca^2+^ signaling may activate PKCs and consequently promote phosphorylation of MOR. PKCα, PKCε, and PKCγ promote the phosphorylation of Serine 363, Threonine 370, and Serine 375 at C-terminal of MOR, which has been implicated in the development of pain and morphine tolerance (Zeitz et al., 2001; Hua et al., 2002; Bjornstrom and Sjoberg, 2005; Newton et al., 2007; Smith et al., 2007; Doll et al., 2011; Bowman et al., 2015). We found that activation of GPER in SH-SY5Y cells promoted membrane translocation of PKCα and PKCε and elevated the expression of phosphorylated-MOR (pMOR), which was negated by the PKC inhibitor Ro 31-8820. These data reveal a novel estrogenic signaling cascade mediated by GPER which ultimately leads to phosphorylation and desensitization of MOR. This signaling mechanism may be relevant to the well-documented gender dymorphisms of pain, morphine analgesia, neuroprotection and cognition as well as the regulation of reproductive behaviors.

Previous studies indicate that the rapid non-genomic estrogenic signaling may also alter gene transcription (Guo et al., 2002; Bjornstrom and Sjoberg, 2005). In a prostate cancer cell (PC-3) line, G-1 may inhibit prostate cancer cell (PC-3) growth, which was mediated through GPER, followed by activation of c-jun/c-fos (Chan et al., 2010). On the other hand, an increase in cytosolic Ca^2+^ may stimulate c-Fos expression, accounting for the neuronal activity-dependent immediate early gene expression and long lasting changes of neural functions (Fukuchi et al., 2015a). Hence we tested whether GPER-mediated Ca^2+^ rise may stimulate c-Fos expression. Indeed either E2 or G-1 stimulated c-Fos expression within 30 min, which was prevented in the presence of the GPER antagonist G15, the IP3R blocker 2APB or the PLC inhibitor U73122. Therefore, GPER-mediated rapid Ca^2+^ signaling may also be transmitted to the nucleus leading to the indirect (i.e., not via the nuclear recetpor, ERα or ERβ) genomic effects.

In the present work, we did not attempt to identify the G protein subtype coupled to GPER. Nevertheless, the profile of the E2/G-1-induced calcium and PKC responses was reminiscent of the Gq-mER-mediated effects previously reported by Qiu et al. (2003) in hypothalamic proopiomelanocortin (POMC) neurons. They showed that E2 rapidly attenuated the potency of GABA_B_ agonist to activate the GIRK current in POMC neurons and the signaling cascade involved Gα_q_-mediated PLC activation upstream of PKCδ, PKA and changes in gene transcription. The effects were mimicked by BSA-conjugated E2 and a non-steroidal compound STX that does not bind ERα or ERβ, leading to the proposal of a Gq-coupled membrane-associated estrogen (STX) receptor (Gq-mER). Although intracellular calcium was not measured in that study, it is conceivable that activation of Gq-mER may also signal through IP3-mediated store calcium release and activation of calcium-dependent PKC isoforms, similarly to the GPER-mediated signaling shown in the current study. The molecular identity of Gq-mER is still unknown. It would be interesting to examine whether Gq-mER and GPER are related or separate mechanisms for rapid estrogenic signaling.

## Conclusion

The present study revealed a novel GPER-mediated estrogenic signaling cascade in neuroblastoma cells. Estrogens may activate the intracellularly located GPER to trigger rapid PLC/IP3-dependent store Ca^2+^ release, which in turn activates PKC isoforms to phosphorylate the μ opioid receptor. The rapid Ca^2+^ signaling may also be transmitted to the nucleus to impact on gene transcription. Such signaling cascade may play important roles in the regulation of opioid signaling in the brain.

## Data Availability Statement

All datasets generated for this study are included in the article/supplementary material.

## Author Contributions

All authors participated in the development of this research and drafting of the manuscript. WR, XS, and GZ made substantial contributions to the conception or design of the work, critically revised the manuscript, supervised the experiments and data analysis, acquired funding, designed the experiments, and analyzed the data. XD, TG, and PG performed the experiments and participated in the analysis of results. YM and LD participated in the immunofluorescence staining experiments. YZ and PL participated in the calcium imaging experiments.

## Conflict of Interest

The authors declare that the research was conducted in the absence of any commercial or financial relationships that could be construed as a potential conflict of interest.
